# An Analysis of Oxycodone and Hydrocodone Distribution Trends in Delaware, Maryland, and Virginia Between 2006 and 2014

**DOI:** 10.7759/cureus.38211

**Published:** 2023-04-27

**Authors:** Conor M Eufemio, Joseph D Hagedorn, Kenneth L McCall, Brian J Piper

**Affiliations:** 1 School of Medicine, Geisinger Commonwealth School of Medicine, Scranton, USA; 2 School of Pharmacy, University of New England, Biddeford, USA; 3 School of Pharmacy, Binghamton University, Johnson City, USA; 4 Medical Education, Geisinger Commonwealth School of Medicine, Scranton, USA

**Keywords:** analgesia, pain, pharmaco-epidemiology, prescription opioids, opioid use disorders

## Abstract

Objective

Opioid medications are widely recognized for their use in analgesia and their addictive properties that have led to the opioid epidemic. Areas with historically high prescribing patterns have been shown to suffer more from the crisis. There is also regional variability in these trends. This study is a county level analysis of oxycodone and hydrocodone use in Delaware, Maryland, and Virginia between 2006 and 2014.

Materials and methods

A retrospective analysis of oxycodone and hydrocodone distributed as collected by the Drug Enforcement Administration’s (DEA) Washington Post Automation of Reports and Consolidated Orders System (ARCOS) in Delaware, Maryland, and Virginia. Raw drug weights in each county were adjusted to “daily average dose” (grams/county population/365) using publicly available population estimates for all state counties. Purchasing data collected from ARCOS was used to compare distribution trends during this period. This study was limited in that ARCOS report quantity of drug distribution rather than average dose of script written.

Results

There was a 57.59% increase in the weight of oxycodone and hydrocodone prescribed between 2006 and 2014. Oxycodone prescriptions increased by 75.50% and hydrocodone by 11.05%. Oxycodone increased across all three states between 2006 and 2010 and declined until 2014. Hydrocodone also increased but to a lesser extent than oxycodone. There was substantial variability in daily average dose of both opioids at the county level in all states. Pharmacies accounted for largest portion of oxycodone (69.17%) and hydrocodone (75.27%) purchased in the region. Hospitals accounted for 26.67% of oxycodone and 22.76% of hydrocodone purchased. Practitioners and mid-level providers, including Nurse Practitioners and Physician Assistants, did not significantly contribute to this increase.

Conclusion

In the states of Maryland, Delaware, and Virginia, the distribution of the prescription opioids oxycodone and hydrocodone increased by 57.59%. Daily average dose increased between 2006 and 2010 in all three states, followed by a decline until 2014. Variability in daily average dose by county highlights the relationship between geography and likelihood of receiving high-dose opioids. Increased monitoring at regional health centers and improving substance abuse treatment infrastructure at the county level may be a more efficient strategy in combating the opioid epidemic. Future research is needed to understand the socioeconomic trends that may influence prescribing trends of opioid medications.

## Introduction

Oxycodone and hydrocodone are the number one and four opioids for annual mean consumption worldwide [1]. Along with other opioid derivatives, these drugs have been widely used in the US inpatient and outpatient facilities for the treatment of both acute and chronic pain. The Food and Drug Administration (FDA) has approved them for moderate to severe pain and, as such, they are regularly prescribed for cancer-related, neurologic, and end-of-life pain [2]. Narcotics are often employed for pain management following surgical interventions. Approximately 51 million Americans undergo inpatient surgery annually. 80% of prescription pain relievers involved either oxycodone or hydrocodone. Patients are often given multiple analgesic medications through different routes and are transitioned to oral opioids at discharge [3]. The rise of the US opioid epidemic has raised concerns about clinicians overprescribing habits and subsequent drug diversion to recreational use by patients. Regulations have sought to address these issues through improved accounting of prescription quantities and dosages administered to patients [4].

Opioids have been documented to increase dependence, especially in the prolonged treatment of chronic pain [2]. The debilitating effects of chronic pain have made opioids one of the most prescribed medication classes in the United States. In 2014, 245 million prescriptions for opioids were filled at retail pharmacies and 3-4% of adults were given long term opioid therapy. Diversion and improper use followed this increased availability. To precede 2014, more than 37% of the 44,000 overdose deaths were attributed to pharmaceutical opioids compared to 19% attributed to street grade heroin [5]. Post-operative pain management interventions are partially to blame for these events. Following procedures, patient’s prescriptions often go unused and are kept in unlocked spaces or are not properly disposed of creating a reservoir for misuse [6].

Numerous studies have demonstrated variability in the prescription of controlled substances by geographic location. Overprescribing was found to be more common in Appalachia, along with southern and western states, with greater variation compared to other modalities of healthcare service use [7]. Additional studies have demonstrated large geographic variability in the treatment of migraines with opioids compared to triptans [8]. Finally, areas with higher prescribing rates have been shown to carry direct correlations with opioid related deaths [9]. Thus, specific analysis is warranted regarding prescribing trends in notable areas including the Mid-Atlantic states of Delaware, Maryland, and Virginia. Despite a similar location along the east coast of the US, their respective sizes, population densities, and regulatory environments differ from one another. In 2018, Delaware attributed 88% of its 401 overdose deaths to opioids. Providers wrote 60.6 opioid prescriptions per 100 residents compared to the national average of 51.4. Maryland providers wrote 45.1 opioid prescriptions per 100 residents and Virginia providers wrote 44.8 [10].

Understanding regional and national opioid prescribing trends is critical to our understanding of the ongoing opioid epidemic and may allow for improved utilization of public resources to resolve these issues. We sought to determine if there are characteristics of these regional counties that may predict if patients are more likely to receive higher average doses. This data was compared to the weight of narcotics purchased by pharmacies, hospitals, practitioners, and mid-level providers in the state.

This manuscript was previously presented as a poster at the 2022 Pennsylvania Pain and Addiction Summit on April 19th, 2022. This report was previously posted to the medRxiv preprint server on July 16th, 2022.

## Materials and methods

This report utilized the US Drug Enforcement Administration's (DEA's) Automated Reports and Consolidated Ordering System (AROCS) comprehensive database to evaluate changes in the use of oxycodone and hydrocodone in Delaware, Maryland, and Virginia between 2006 and 2014, the time period for which county-level data is currently available. Total weights of each medication were examined by county in each state. Data was extracted from the DEA's Washington Post ARCOS database which reports the monthly and quarterly weights of controlled substances prescribed along with purchasing data from distributors [11]. This included total grams of oxycodone and hydrocodone prescribed in Delaware, Maryland, and Virginia from 2006 to 2014. This resource has been frequently used in prior pharmacoepidemiologic studies [12-14]. The main units of this investigation may be less familiar than others such as "number of prescriptions". Prior studies have indicated that examining the total weight of oxycodone in this database relative to reports in a Prescription Drug Monitoring Program revealed a high correlation (R = .99) [12].

ARCOS data is limited to raw weights distributed to providers and does not include statistics on the number of patients receiving opioid therapy or the average weight and duration of each prescription. To approximate the changes in each counties relative use, the total weight of drug prescribed by county was divided by the county’s population (grams/population/365). The raw weights were adjusted to morphine equivalent weights (hydrocodone = 1.5 g*{reported weight} and oxycodone= 1 g*{reported weight}). County populations were obtained from the US Census Bureau; however, inconsistencies were noted in the years of reporting in some counties [15]. Missing data was substituted with population statistics from the St. Louis Federal Reserve which revealed similar population trends to those already established [16]. This yielded a standardized dosing comparison on which further evaluation could be completed. Heat maps were generated using Someka Excel software (Someka, İzmir, Turkey). Counties were graded according to how many times greater than their states mean they deviated during their periods of increased opioid use. Poverty rates were extracted from the St. Louis Federal Reserve’s database for 2010 which were compared to daily average dosing changes in high prescribing counties.

An examination of states specific buyer data was undertaken using reports from the DEA's ARCOS. Reports including yearly weight purchased were used to compare the rates of distribution from different healthcare entities. This system tracks controlled substances distributed to pharmacies, hospitals, practitioners, and mid-level providers nationwide. Data was sorted by our states of interest. The Institutional Review Board at the University of New England and Geisinger exempted this study from ethical considerations.

## Results

**Figure 1:**
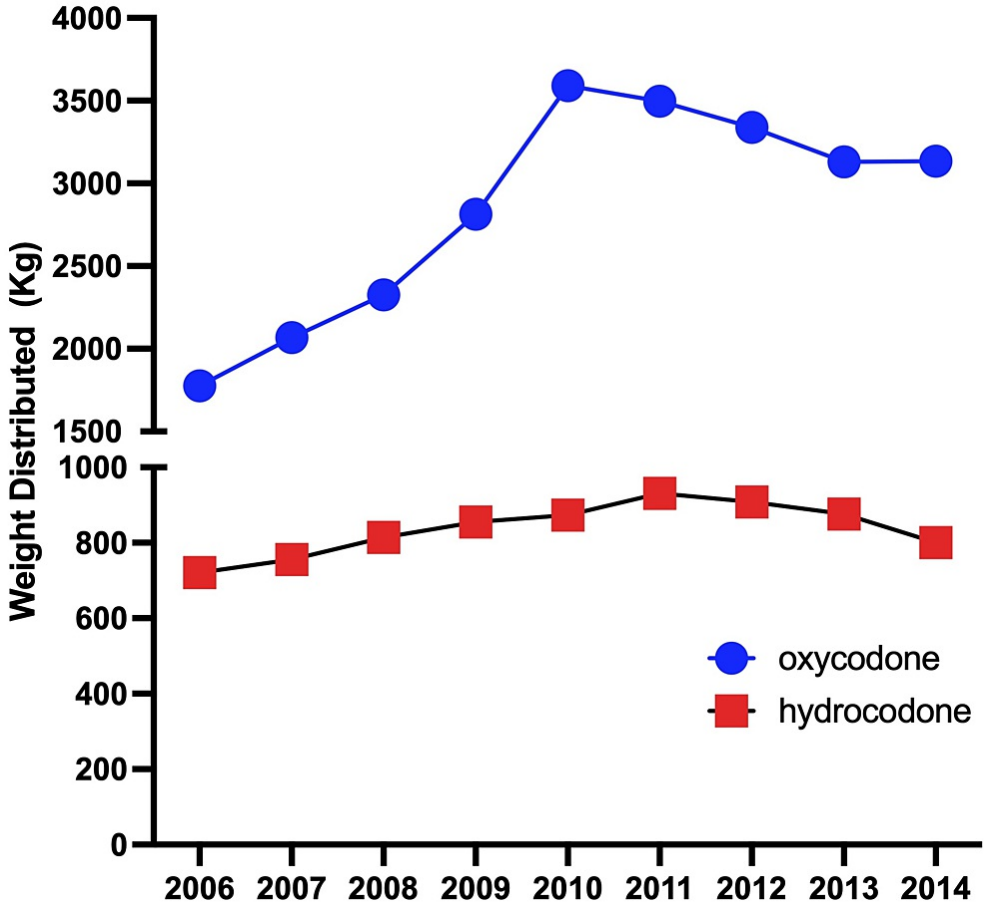
Trends in oxycodone and hydrocodone weights prescribed in Delaware, Maryland, and Virginia (2006-2010)

Oxycodone use in Delaware, Maryland, and Virginia increased between 2006 and 2014 by 76.5%, and hydrocodone use increased by 11.05%. Overall, this yielded a total increase of 57.59% (t=1.0394, p=0.3573, Figure 1).

**Figure 2:**
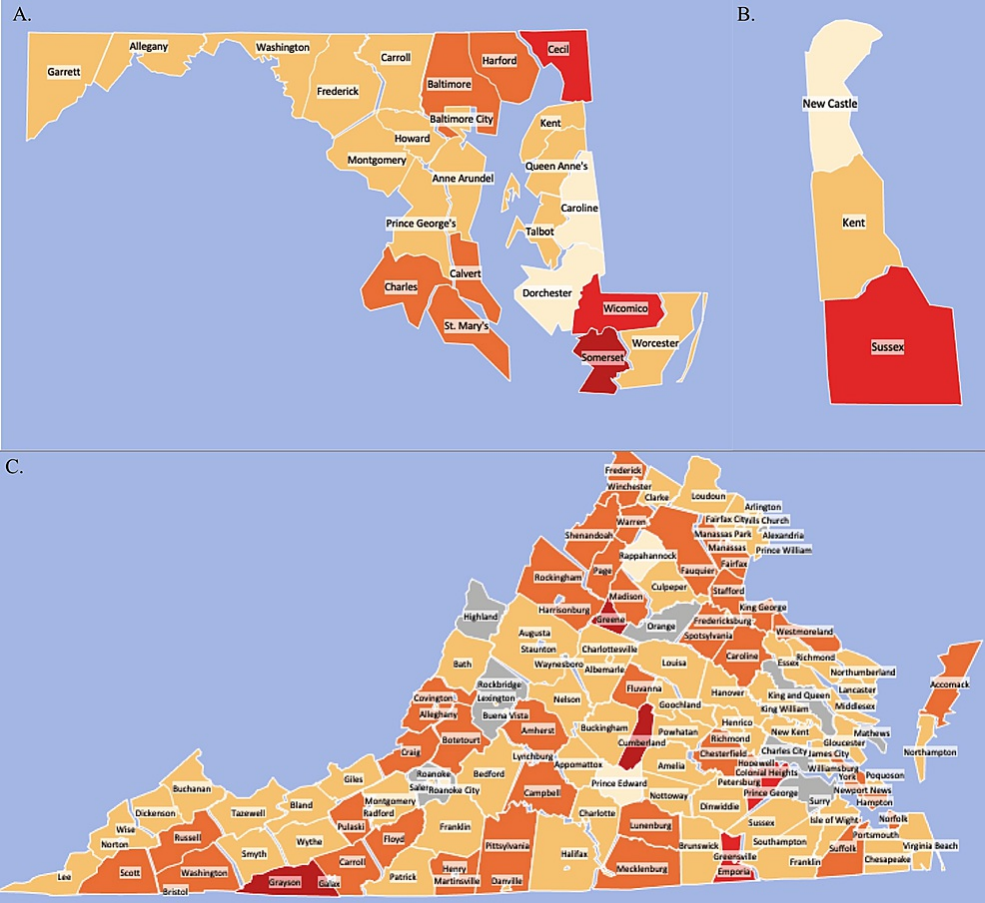
% Change in daily oxycodone weight prescribed by county as reported by ARCOS (2006-2010) A. % change of daily average oxycodone weight prescribed in Maryland by county (2006-2010) B. % change of daily average oxycodone weight prescribed in Delaware by county (2006-2010) C. % change of daily average oxycodone weight prescribed in Virginia by county (2006-2010) *Darker Red = More standard deviations above the state mean; *Grey = Population data not available; ARCOS = Automation of Reports and Consolidated Orders System

Maryland’s statewide average daily oxycodone dose increased 84.51% between 2006 and 2010 (t=3.6593, p<0.05). Daily average doses continued increasing through 2011. There was substantial variability between counties as indicated by Figure 2. A pattern of higher per capita usage was noted around northern Baltimore suburban counties, and along both sides of the southern Chesapeake region. Somerset county presented the largest increase in daily average dose and a 213.45% increase between 2006 and 2010 (t=3.3156, p<0.05). High increases in usage were also observed in Cecil (+206.90%, t=3.1725, p<0.05), and Wicomico (+156.16%, t=2.0114, p=0.0791). These counties lie in the along the border with Delaware, with Wicomico and Somerset are located close to Sussex County (Figure 2A). There was an increase in oxycodone distribution within the region between 2006 and 2010 (t=1.8317, p=0.1409). In Delaware, daily average dosages increased by 154.38% (t=2.50, p=0.0666). Daily dosages in Kent County accounted for +146.70% (t=1.9654, p=0.0670) change and Sussex County showed a significant +185.44% change (t=2.1461, p<0.05). New Castle also followed this trend with a 111.98% (t=5.5367, p<0.05) increase. Kent (12.80%) and Sussex (14.12%) Counties were above the state average (11.90%) for poverty rates (Figure 2B). Virginia’s statewide daily average dosage of oxycodone increased by +52.96% (t=3.7637, p<0.05) between 2006 and 2010. However, the largest year over year increase came from the 2010-2011 reporting year (+18.15%). Several counties presented with increases greater than two standard deviations above Virginia’s state average, including Greene (+1272.50%) (t=7.4650, p<0.05), Grayson (+400.60%, t=9.4350, p<0.05), and Cumberland (+384.10%, t=12.6511, p<0.05). Greene county had a poverty rate of 9.7% compared to 11.1% of Virginia residents in 2010. Like Maryland, Virginia showed substantial variability among counties as evidenced by the Figure 2. There do not appear to be significant correlates between geographic location and increase in daily average dose in Virginia. Many counties in northern Virginia increased in daily average dose above the state mean between 2006-2010, but not above a standard deviation. Counties with similar increases were seen in closer proximity to the West Virginia border or central Virginia as opposed to the coastal regions (Figure 2C).

**Figure 3:**
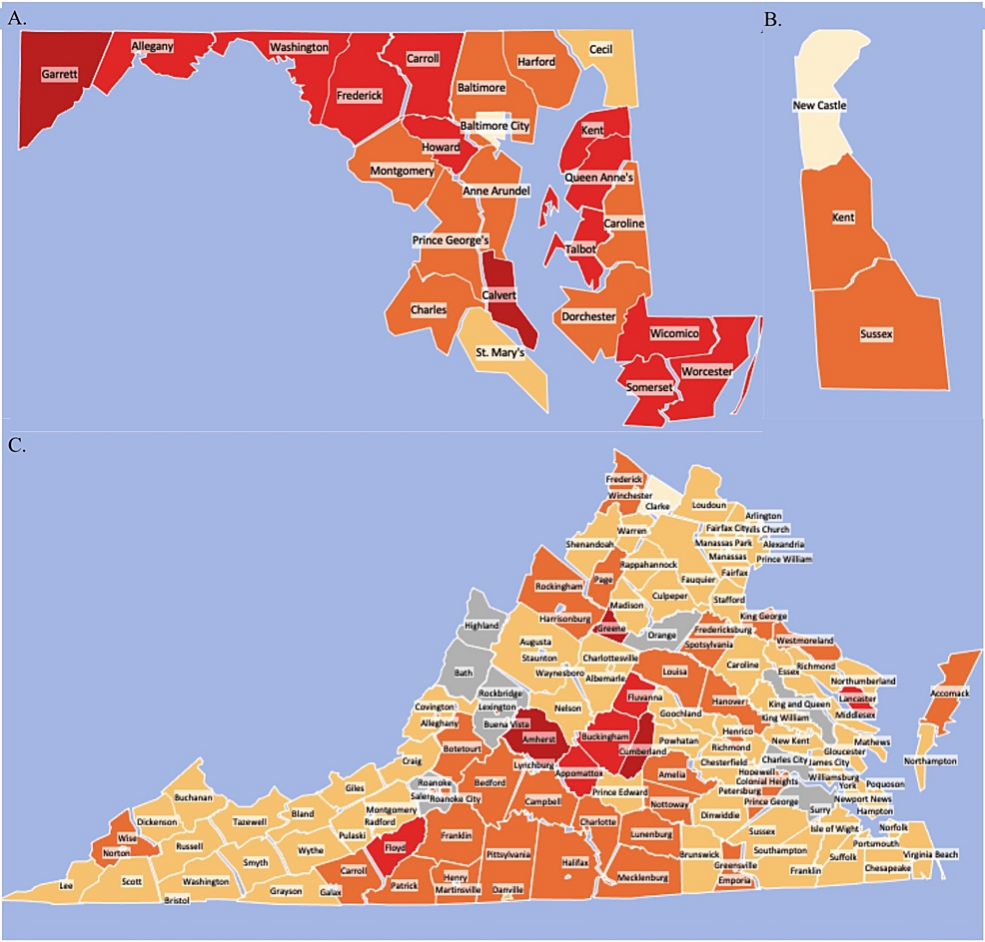
% Change in hydrocodone MME by county as reported by ARCOS (2006-2010) A. % change in daily average hydrocodone weight prescribed in Maryland by county (2006-2010) B. % change in daily average hydrocodone weight prescribed in Delaware by county (2006-2010) C. % change in daily average hydrocodone weight prescribed in Virginia by county (2006-2010) *Darker Red = More standard deviations above state mean; *Grey = Population data not available; ARCOS = Automation of Reports and Consolidated Orders System; MME = Morphine Milligram Equivalents

Trends in hydrocodone use remained steady across all three states. Most Maryland counties showed a decrease in daily average dose but there were significant increases in Garrett (+79.36%; t= 2.2892, p=0.05), Allegany (+33.18%; t=11.0918, p<0.05), Washington (+32.70%; t=4.6917, p=0.05), and Frederick (+31.39%; t=9.9558, p<0.05) counties, with Garrett (15.1%) having a poverty rate above Maryland's 2010 rate (9.90%). The most significant reduction occurred in Baltimore City (-77.11%; t=7.2183, p<0.05). Maryland showed a slightly increased usage (+4.82%) between 2006 and 2014 (Figure 3A). Delaware decreased its average daily dose by -14.91% between 2006 and 2014 (t=1.0162, p=0.3670). Both Kent (-20.06%; t=20.5349, p<0.05) and New Castle (-29.36%; t=21.6495, p<0.05) decreased their daily average dosage with only Sussex County (+4.69%; t=16.5075, p<0.05) showing an increase in distribution during this time (Figure 3B). However, Virginia showed an overall increase of +36.52% in daily average dose between 2006 and 2014. Many of the contributing counties were in central and southern regions of the state and appear in clusters. Amherst (13.60%) and Cumberland (17.20%) were two counties with above average poverty levels and use greater than two standard deviations above the state mean. There was significant variability between Virginia counties. When considering only the counties that increased their daily average hydrocodone dose, there was a 240-fold difference between the lowest (+2.36%) and highest (+568%) increase (Figure 3C).

**Figure 4:**
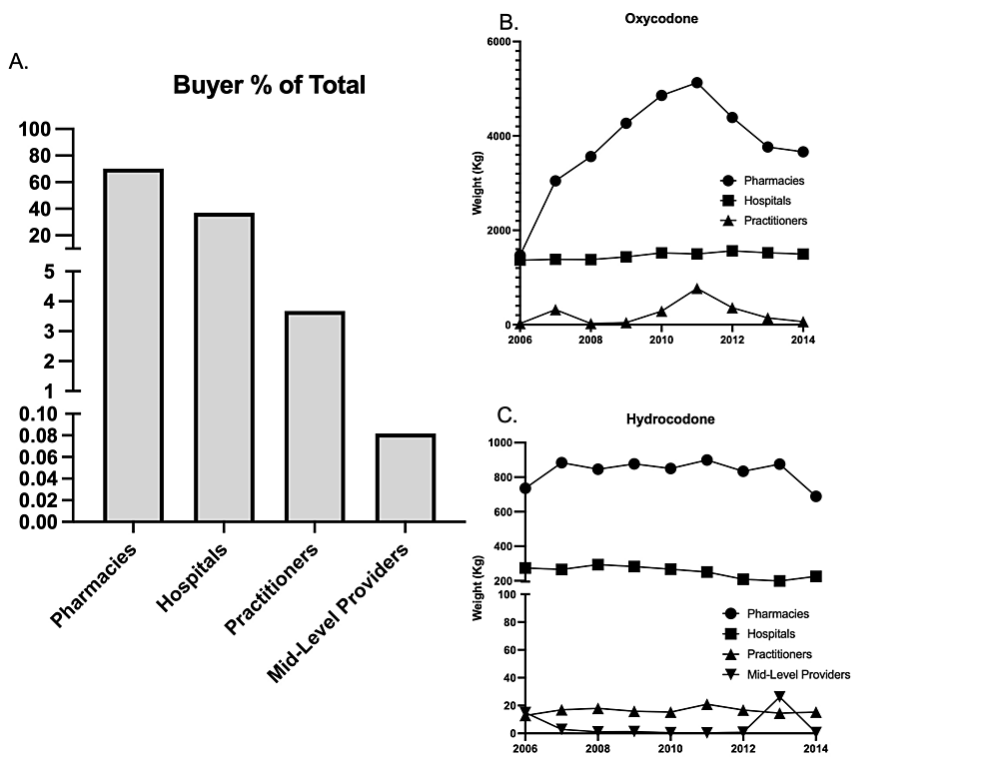
Buyer trends for oxycodone and hydrocodone in Delaware, Maryland, and Virginia A. % of total opioid bought by pharmacies, hospitals, practitioners, and mid-level providers B. Trends in oxycodone purchasing (2006-2014) C. Trends in hydrocodone purchasing (2006-2014)

ARCOS catalogues the weights of controlled substances bought by pharmacies, hospitals, practitioners, and mid-level providers. Pharmacies were responsible for purchasing over two-thirds of oxycodone (69.17%; t=3.1698, p<0.05) and three-quarters of hydrocodone (75.27%, t=7.9568, p<0.05) (Figure 4A). Between 2010 and 2011, pharmacies purchased +248.27% (t=7.3787, p<0.05) more oxycodone (Figure 4B) and +22.18% (t=12.5537, p<0.05) more hydrocodone (Figure 4C). Hospitals followed in both oxycodone (+26.67%) and hydrocodone (+22.76%) purchasing. Practitioners increased the amount of oxycodone (+3,170.37%) and hydrocodone (+63.12%) between 2006-2011. However, this portion of the total, 4.12% of oxycodone and 1.47% of hydrocodone, did not appreciably affect the cumulative trends. Mid-level providers only accounted for 0.49% of hydrocodone purchased between 2006-2014. Between 2010 and 2011, this decreased by -98.12%.

## Discussion

This study investigated trends in oxycodone and hydrocodone weights prescribed in the US states of Delaware, Maryland, and Virginia between 2006 and 2014, and indicated a 53.59% increase in the weight of oxycodone distributed. Trends increased year over year until 2010 followed by a period of decline to 2014 levels. Analysis for total weight and daily average dose (adjusted for population) showed a comparable increase in oxycodone usage with a relatively small change (+11.05%) in hydrocodone usage.

Reduction in use following peak years may be reflective of new data that has come to light involving the relationship between opioid dose and risk of overdose [17]. Regulatory intervention likely influenced practitioners to adjust prescribing habits across the US. The peak year for opioid prescriptions during our period of interest in the US occurred between 2010 and 2011 in adjusted Morphine Milligram Equivalents (MME) form. Additional data from the CDC reported that between 2006 and 2015, MME peaked across all opioid classes at 782 mg per capita followed by a gradual decrease which still left per capita prescribing weights three times greater than in 1999 [18].

Differences in distribution patterns related to geography and demographic factors have been the subject of research since the institution of prescription monitoring programs. Overall, studies have shown that counties located in the Appalachia region along with southern and western states are more susceptible to high numbers of prescriptions per person. These statistics are correlated with the larger county populations and proportions of residents who identify as non-Hispanic/African American or are poor, uninsured, and living in urban areas [7]. The percentage of residents living in poverty in 2010 were examined and compared to oxycodone weight prescribed between 2010 and 2011. During this period, the national poverty rate stood at 15.3%. 9.7% of Greene County, Virginia, residents reported living below the poverty line. An appreciable portion of residents in Mecklenburg (20.2%) and Southampton (16.4%) were living below the poverty line. The Virginia state poverty rate was 11.1% of residents. With two out of three of the highest prescribing counties above average poverty rates, this seems to be an indicator of likelihood of high daily average oxycodone dosage during this spike. The percentage of Delaware residents living in poverty was estimated to be 11.9% in 2010. Kent (12.80%) and Sussex (14.12%) were the two counties with above average poverty rates and the only counties where the weight of oxycodone prescribed had increased. However, New Castle’s reported poverty rate was a 11.2% and demonstrated a comparatively moderate increase of 4.40% in usage. Thus, in Delaware the poverty status of residents does not necessarily predict oxycodone dosage. The same is true for Maryland (MD). Frederick, MD (5.6%) and Harford, MD (6.9%) both had poverty rates lower than 2010 state average (9.9%) despite reporting large increases in oxycodone dosage. It has been noted that within a geographic region, the quantity of opioids, specifically hydrocodone, prescribed to patients undergoing the same general surgical procedure varied by provider [19]. Although poverty may be a factor for the dosage of a prescription, this is likely confounded by other factors.

Delaware’s overall daily average dose of hydrocodone decreased during our study period. The only county to show an increased dose was Sussex where 14.12% of the population was reported to live below the poverty line in 2010. However, the daily average dose of hydrocodone remained within one standard deviation above the state average, similar to Kent County. Though similar state trends were observed in Maryland, Garrett and Allegany counties represented two areas of increased hydrocodone use. Only Garrett County would suggest a relationship between poverty status and hydrocodone use in the state, as Calvert’s poverty status was close to the state average. Virginia was the only state noted as having increased hydrocodone use during our study period but had significant county wide variability in dose. Two notable counties were Amherst and Cumberland, where hydrocodone use increased above 2 standard deviations. Both showed higher than average poverty levels. These findings may indicate that poverty is not a predictive measure to predict areas of higher than average hydrocodone use accurately.

Additionally, we sought to explore changes in oxycodone distribution methods by evaluating ARCOS buyer data. ARCOS maintains yearly records of controlled substances purchased by pharmacies, hospitals, practitioners, mid-level providers, and teaching institutions across the US. Understanding opioid distribution patterns is key in designing protocol aimed at limiting demand in areas at high risk of overprescribing. Many states have implemented laws in recent years to strictly regulate the standards for prescribing opioids and other scheduled substances. In Delaware, the “Uniform Controlled Substances Act” places clear definitions on administration and dispensing while also laying designating prescribers towards which the law applies including physicians, dentists, and nurse practitioners. As defined by this law, all prescriptions, must be verified by a licensed provider regardless of circumstance. For example, emergent prescriptions must be verified by a board-certified physician [20].

Similar laws in other Mid-Atlantic states apply to prescribers and dispensers. Virginia does not allow for a narcotic supply exceeding seven days which also applies to prescriptions given on emergency department discharge. Providers can issue a 14 day supply of opioids following a surgical procedure. The state law also puts forth specific measures to be taken with high doses or recurrent use. Prescriptions above 120 MME require reasonable justification or a pain management referral. Naloxone is also prescribed to patients with risk factors including a prior overdose, substance misuse disorder, or concomitant benzodiazepine use [21]. In Maryland, doses >50 MME require an increase in follow up frequency and for naloxone to be offered to address overdose concerns. Pain management is to be consulted with doses >90 MME [22]. It should be noted that prior ARCOS research has found that prescribing laws had no appreciable impact in Maryland and Delaware [23].

With emphasis on provider data, similar interest was taken in entities purchasing controlled substances from manufacturers. Within the region, pharmacies were the largest buyers of both oxycodone (69.2% of total) and hydrocodone (75.3% of total). This represented 70.2% of the weight of opioids bought between 2006 and 2014. Hospitals were the second largest buyer of oxycodone (26.7%) and hydrocodone (22.8%). Year-to-year trends showed that oxycodone purchases substantially increased between 2006 and 2011 between pharmacies. In the peak year for average oxycodone dosage, 2011, pharmacies increased purchasing by +248.3% from 2006, compared to a +9.64% increase in hospital purchasing. This substantial increase likely associated with increased demand for outpatient pain therapy. These trends fall in line with national analysis by the FDA which found an average increase of 15 billion MME per year supplied to retail pharmacies [24]. ARCOS data provided limited information on the type of pharmacy in question. This complicates trend analysis when evaluating changes in corporate vs independent pharmacy distribution patterns. Delaware saw a 95% increase in the number of independent pharmacies operating in the state between 2010 (19) and 2019 (37) which was the largest increase in the United States during this period. Smaller increases were seen in Maryland (+36.0%) and Virginia (+8.0%) [25].

There was additional interest in purchasing trends of practitioners and mid-level providers. In total, practitioners in Delaware, Maryland, and Virginia, increased oxycodone purchase weight (+3170.40%) and hydrocodone weight (+63.10%) between 2006 and 2011. This describes 4.11% and 1.47% of purchase weights between the two medications, respectively. Consistent data for mid-level providers was only available for hydrocodone and represented a decrease of -98.1% during peak years accounting for <1% of hydrocodone purchased. Mid-Level providers, specifically Nurse Practitioners (NP) and Physician’s Assistants (PA), are increasingly autonomous in key areas of clinical care. In 2006, they accounted for 12.7% of emergency department patients seen, which was an increase from 5.5% in 1997 [26]. In all three states of interest, NPs and PAs are permitted to prescribe and dispense samples of Schedule II-V substances. NPs are not allowed to procure in Delaware and Maryland while Virginia is the only one that allows NPs to do so [27].

National studies have suggested that NPs/PAs prescribed opioids in similar patterns to primary care physicians, yet were more likely to give high-frequency high-dose regimens [28]. Though procurement by mid-level providers was evaluated in this study, the possibility that mid-level providers are writing for opioid regimens in hospital settings cannot be discounted. With the US ranking third for per capita consumption of prescription opioids [1], further data to guide opioid stewardship programs are needed.

Thorough investigation is also needed to evaluate the relationship between diagnosis and opioid prescriptions, particularly in rural areas that saw the highest population adjusted usage in these states. It was noted that counties with lower population densities saw the most significant increases in dosage by percent in their respective states. This finding may indicate the need for more specific investigations into the clinical use of opioids in rural health centers to confirm other studies. For example, previous analysis has suggested that there was an undertreatment of cancer-related pain in rural areas of Virginia in 2015 [29]. ARCOS data does not catalogue International Statistical Classification of Diseases and Related Health Problems-10 (ICD-10) codes and therefore conclusions about prescriber behavior could not be drawn on the case of diagnostic trends. However, this information is imperative for identifying the prescribing habits of providers and improving these trends in the interest of public health and patient safety. Other variables such as access to specialized oncologic care, diagnostic rates of terminal cancer, and local attitudes toward terminal pain treatment should also be considered. Therefore, future studies, drawn from smaller cohorts, could analyze the relationship between geography, conditions, and use of opioids.

There are several limitations to this pharmacoepidemiologic study. Although ARCOS catalogues weights of controlled substances, it does not present information regarding the total number of opioid prescriptions or the average dosage of each regimen. Using population adjusted weights prescribed in each county was the method for this study; however, more conclusive evidence may come from additional information on the number of patients receiving controlled substances. Our calculations may also be impacted in less populated counties. We attempted to evaluate trends by looking at percentage changes over a given period; however, this may not be enough to predict whether prescriptions are abnormally high or if there is a skew in the data from specific counties. Population data is also subject to reporting inconsistencies between the US Census Bureau and the St. Louis Federal Reserve. Additionally, information given on mid-level procurement weights is done only by practicing entity and not as part of any larger system. Therefore, we cannot accurately determine the prescribing trends in mid-level settings and further investigations could seek comprehensive data on frequency and average dose given by providers. This study is also limited to the reporting period of the ARCOS database. With the development of stringent reporting standards, daily average dosing for all opioid medications with surely change. There is the need to compare the data found in this study with reporting from the current decade to evaluate the effects of these regulatory reactions to opioid overuse.

Overall, this analysis demonstrates three contiguous states with significant variability in the prescription of oxycodone and hydrocodone between their counties. These findings imply the need for federal, state, and local resources and policies to limit the use of these medications to address the opioid epidemic. This variability suggests that resources from state and local organizations be handled by county authorities and health systems as opposed to regional ones. Given the differences in resources and demographics within these states, an improved substance abuse protocol and treatment infrastructure at county levels, as opposed to state measures, may be a more effective strategy for combating misuse. A concern in the scope of pharmacoepidemiology remains the use of illicit opioids. Further research is needed to quantify the regional relationship between prescribing habits and progressive dependence on opioid derivatives, such as heroin and fentanyl, to continuously modify policies in the face of this growing substance use crisis.

## Conclusions

In conclusion, this report identified increases in the distribution of oxycodone and hydrocodone in Delaware, Maryland, and Virginia between 2006 and 2014 using ARCOS data. Examination revealed a combined 57.59% increase in weight between the two. Peak years for high dosage occurred between 2006-2010, representing a substantial rise in the daily average opioid dose in the region. Further investigation revealed that pharmacies were the largest suppliers of both substances, followed by hospitals. Practitioners and mid-level providers also increased the weight of opioids bought during this study period. These points in the opioid distribution chain should be evaluated as areas to further regulate in addressing inappropriate prescription usage. Further state-level investigations are needed to understand better the economic and social factors influencing prescription trends and the effects of geography on substance overuse.

## References

[1] Richards GC, Aronson JK, Mahtani KR, Heneghan C (2022). Global, regional, and national consumption of controlled opioids: a cross-sectional study of 214 countries and non-metropolitan territories. Br J Pain.

[2] Baldini A, Von Korff M, Lin EH (2012). A review of potential adverse effects of long-term opioid therapy: a practitioner's guide. Prim Care Companion CNS Disord.

[3] Hah JM, Bateman BT, Ratliff J, Curtin C, Sun E (2017). Chronic opioid use after surgery: implications for perioperative management in the face of the opioid epidemic. Anesth Analg.

[4] (2022). Addressing Problematic Opioid Use in OECD Countries. https://www.oecd.org/health/addressing-problematic-opioid-use-in-oecd-countries-a18286f0-en.htm.

[5] Volkow ND, McLellan AT (2016). Opioid abuse in chronic pain--misconceptions and mitigation strategies. N Engl J Med.

[6] Bicket MC, Long JJ, Pronovost PJ, Alexander GC, Wu CL (2017). Prescription opioid analgesics commonly unused after surgery: a systematic review. JAMA Surg.

[7] McDonald DC, Carlson K, Izrael D (2012). Geographic variation in opioid prescribing in the U.S. J Pain.

[8] Lee JH, Shewale AR, Barthold D, Devine B (2021). Geographic variation in the use of triptans and opioids for the acute treatment of migraine attacks. Headache.

[9] Gomes T, Juurlink D, Moineddin R, Gozdyra P, Dhalla I, Paterson M, Mamdani M (2011). Geographical variation in opioid prescribing and opioid-related mortality in Ontario. Healthc Q.

[10] (2022). U.S. State Opioid Dispensing Rates, 2018. https://www.cdc.gov/drugoverdose/rxrate-maps/index.html.

[11] (2022). Automation of Reports and Consolidated Orders System (ARCOS). https://www.deadiversion.usdoj.gov/arcos/.

[12] Piper BJ, Shah DT, Simoyan OM, McCall KL, Nichols SD (2018). Trends in medical use of opioids in the U.S., 2006-2016. Am J Prev Med.

[13] Simpson KJ, Moran MT, Foster ML (2019). Descriptive, observational study of pharmaceutical and non-pharmaceutical arrests, use, and overdoses in Maine. BMJ Open.

[14] Vaddadi SM, Czelatka NJ, Gutierrez BD, Maddineni BC, McCall KL, Piper BJ (2021). Rise, and pronounced regional variation, in methylphenidate, amphetamine, and lisdexamfetamine distribution in the United States. PeerJ.

[15] (2022). US Census Bureau. https://www.census.gov/.

[16] (2022). Federal Reserve Bank of St. Louis. https://fred.stlouisfed.org/.

[17] Bohnert AS, Valenstein M, Bair MJ, Ganoczy D, McCarthy JF, Ilgen MA, Blow FC (2011). Association between opioid prescribing patterns and opioid overdose-related deaths. JAMA.

[18] Guy GP Jr, Zhang K, Bohm MK (2017). Vital signs: changes in opioid prescribing in the United States, 2006-2015. MMWR Morb Mortal Wkly Rep.

[19] Hill MV, McMahon ML, Stucke RS, Barth RJ Jr (2017). Wide variation and excessive dosage of opioid prescriptions for common general surgical procedures. Ann Surg.

[20] (2022). Uniform Controlled Substances Act Regulations. https://regulations.delaware.gov/AdminCode/title24/Uniform%20Controlled%20Substances%20Act%20Regulations.shtml/.

[21] (2022). Virginia Law Administrative Code. https://law.lis.virginia.gov/admincode/title18/agency150/chapter20/section174/.

[22] (2022). Maryland Board of Physicians Board Guidance. https://www.mbp.state.md.us/resource_information/res_con/resource_consumer_od_board_guidance.aspx.

[23] Davis CS, Piper BJ, Gertner AK, Rotter JS (2020). Opioid prescribing laws are not associated with short-term declines in prescription opioid distribution. Pain Med.

[24] (2022). FDA Analysis of Long-Term Trends in Prescription Opioid Analgesic Products: Quantity, Sales, and Price Trends. https://www.fda.gov/files/about%20fda/published/FDA-Analysis-of-Long-Term-Trends-in-Prescription-Opioid-Analgesic-Products--Quantity--Sales--and-Price-Trends.pdf.

[25] (2022). Independent Pharmacies in the U.S. are More on the Rise than on the Decline. https://www.pcmanet.org/wp-content/uploads/2020/03/FINAL_Independent-Pharmacies-in-the-U.S.-are-More-on-the-Rise-than-on-the-Decline.pdf.

[26] Brown DF, Sullivan AF, Espinola JA, Camargo CA Jr (2012). Continued rise in the use of mid-level providers in US emergency departments, 1993-2009. Int J Emerg Med.

[27] DEA Diversion Control Division. (2022). Mid-Level Practitioners Authorization by State. https://www.deadiversion.usdoj.gov/drugreg/practioners/mlp_by_state.pdf.

[28] Lozada MJ, Raji MA, Goodwin JS, Kuo YF (2020). Opioid prescribing by primary care providers: a cross-sectional analysis of nurse practitioner, physician assistant, and physician prescribing patterns. J Gen Intern Med.

[29] LeBaron VT, Camacho F, Balkrishnan R, Yao NA, Gilson AM (2019). Opioid epidemic or pain crisis? Using the Virginia All Payer Claims Database to describe opioid medication prescribing patterns and potential harms for patients with cancer. J Oncol Pract.

